# Advanced Marine Predator Algorithm for Circular Antenna Array Pattern Synthesis

**DOI:** 10.3390/s22155779

**Published:** 2022-08-02

**Authors:** Eunice Oluwabunmi Owoola, Kewen Xia, Samuel Ogunjo, Sandrine Mukase, Aadel Mohamed

**Affiliations:** 1 School of Electronics and Information Engineering, Hebei University of Technology, Tianjin 300401, China; bunmso@gmail.com (E.O.O.); ssandrinem@gmail.com (S.M.); 2 Department of Physics, Federal University of Technology, Akure 340271, Ondo State, Nigeria; stogunjo@futa.edu.ng; 3 School of Mechanical Engineering, Hebei University of Technology, Tianjin 300401, China; aadelm380@gmail.com; 4 Department of Mechanical Engineering, University of Nyala, Nyala 11111, Sudan

**Keywords:** advanced marine predator algorithm, beam pattern, circular antenna array, sidelobe level

## Abstract

The pattern synthesis of antenna arrays is a substantial factor that can enhance the effectiveness and validity of a wireless communication system. This work proposes an advanced marine predator algorithm (AMPA) to synthesize the beam patterns of a non-uniform circular antenna array (CAA). The AMPA utilizes an adaptive velocity update mechanism with a chaotic sequence parameter to improve the exploration and exploitation capability of the algorithm. The MPA structure is simplified and upgraded to overcome being stuck in the local optimum. The AMPA is employed for the joint optimization of amplitude current and inter-element spacing to suppress the peak sidelobe level (SLL) of 8-element, 10-element, 12-element, and 18-element CAAs, taking into consideration the mutual coupling effects. The results show that it attains better performances in relation to SLL suppression and convergence rate, in comparison with some other algorithms for the optimization case.

## 1. Introduction

Antenna arrays have many applications in communications systems, radars, medical imaging, remote sensing, Internet of Things (IoT), and signal processing because of their ability to provide high directive characteristics [1], spatial diversity [2], adaptive beamforming, and beam steering [3]. The effectiveness of antenna arrays is highly dependent on their geometry, the number of antennas, element spacing, beam pattern, and sidelobe level (SLL). To meet the necessities of long-distance communication, antenna arrays with very directional qualities are required in many applications [4]. To deal with the high free-space route loss problem of millimeter-wave (mmWave) transmissions and fulfill the increasing capacity demand, the fifth-generation (5G) cellular communication technology requires large-scale antenna array systems [5]. The urgency to meet the demand for qualitative and quantitative energy has necessitated vast research into improving the efficiency of antenna arrays, in the aspect of SLL reduction, interference nulling, narrow directivity, and high gains.

Many research works have been dedicated to developing techniques for the pattern synthesis of antenna arrays with a narrow main lobe and reduced sidelobe level [2,6]. Pattern synthesis of different types of antenna such as linear array, circular array, hexagonal array, concentric circular array, time modulated arrays, and many more using some heuristic algorithms has been carried out by several researchers [7,8,9,10,11]. To solve the antenna array optimization, the choice of the optimization algorithm is essential, as is the objective function capturing the designated relevance of the antenna array. Mutual coupling is likewise a relevant factor to consider in the design of the antenna array, for it has a great effect on the field performance of the antennas. Thus, recent research work carefully put into consideration the mutual coupling effect during the antenna array synthesis [7,9,12].

Over the years, the addition of refined or optimal parameters and hybridization of multiple metaheuristic algorithms has been an effective way of improving the efficiency of the algorithms combined for antenna array synthesis. Ref. [13] applied an invasive weed optimization algorithm to the synthesis of a rectangular array, circular arrays, and the bee-hive. The modification of the IWO algorithm for the optimization of time-modulated LAA was proposed by Basak et al. [14]. The IWO was improved by including two parallel populations and a new routine of changing the mutation step-size with iterations, which made the algorithm achieve better performance. Jang et al. performed the circular lattice array structure optimization in aperture synthesis radiometers using particle swarm optimization (PSO) [15]. Hosseini and Jafarian [16] came up with the concept of hybridizing invasive weed optimization and particle swarm optimization (HIWOPSO) so that IWO will benefit from the swarm intelligence of PSO and avoid being trapped in local solutions. A hybrid invasive weed optimization (IWO) and wind driven optimization (WDO) was also proposed and adopted for the synthesis of uniformly spaced LAA and non-uniform CAA by Mahto and Choubey [17].

The optimization of a circular array using efficient biogeography-based optimization (EBBO) was proposed by ref. [18]. The authors improved the standard BBO and applied it to optimize jointly the amplitude current and element position of the CAA. A convex improved genetic algorithm (CIGA) was proposed by Li et al. [19] for the beam pattern synthesis of sparse arrays. This hybrid algorithm was able to adjust the excitation and positions of the arrays to suppress the peak SLL and obtain better results as compared to some other existing algorithms. The least mean square (LMS) algorithm and sample matrix inversion (SMI) were also combined to optimize the weight of the adaptive antenna array at different arrival angles [20]. Ref. [21] introduced the use of a hybrid cuckoo search algorithm with convex programming (CS–CP) to solve the sparse LAA based on a subarray scheme synthesis problem. In each subarray, the number, excitation, and spacing are optimized with the minimum or maximum spacing constraints. The optimized array was able to achieve an improved peak SLL and reduced excitation control number. Wei et al. [22] proposed a hybrid artificial neural network (ANN) and convex optimization for linear and planar antenna array design, taking into consideration the coupling effect between the antenna elements. The ANN was applied to design and optimize the radiation pattern and element positions of the array antenna, whereas the convex optimization was employed for the synthesis of the main lobe direction and SLL of the array.

An improved differential evolution (DE) algorithm combined with the successful-parent-selecting (SPS) framework, named SPS-JADE, was proposed and adopted for the pattern synthesis of LAA by Zhang et al. [23]. In this algorithm, the SPS framework was used to mitigate the stagnation problem of DE algorithms, and hence improved the overall performance of the algorithm in terms of the global searchability, convergence rate, and robustness. To the synthesis of LAA using GWO [24], Lakhlef et al. [25] proposed a modified GWO by introducing the competitive exclusion selection inspired from the genetic algorithm in the proposed algorithm with the inclusion of Gaussian function. However, a Jaya-grey wolf optimizer was suggested for the design of the antenna array for 5G communications systems [26]. Both GWO and the Jaya algorithm have the advantage of only two parameters setting; therefore, the features of both algorithms are aggregated to form a new hybrid, which proved to be effective.

The marine predator algorithm (MPA) [27] has recently been proposed for solving optimization problems of various kinds. The MPA optimization method has been applied to solving some engineering problems such as fault diagnosis of rolling bearing [28] and the PID tuning problem for DC motor speed control [29], but it is yet to be adopted in solving antenna synthesis problems. Therefore, this work introduces the MPA to the electromagnetic world both in its standard form and also in its improved form. Research has proven the standard MPA to exhibit deficiency in terms of the inability to produce a diverse initial population with high productivity, slow escape from the local optimum, and a little exploration of the search space [29,30]. We propose a novel algorithm called the advanced marine predator algorithm (AMPA) for better optimization performance in solving engineering problems, most especially in the electromagnetic field. AMPA’s velocity update strategy aids MPA in overcoming the problem of slow escape from the local optimum and increases the algorithm’s convergence rate.

The major contributions of the work are as follows:

1. Introduction of an improved velocity (stepsize) update strategy and an adaptive parameter to control the velocity of the prey, thus improving the exploration capability and mitigating the stagnancy problem of MPA.
2. A chaotic sequence called the Chebyshev map is added to the prey update to aid the stepsize and thus enhance the exploitation ability of the algorithm. These improvements effectively help MPA to overcome the problem of being stuck easily in local optimum, as well as improve the convergence rate of the overall algorithm.
3. We formulated the peak SLL suppression optimization problem for the synthesis of CAA and applied the novel AMPA to solve the objective functions. The excitation current and the inter-element spacing of four examples of CAA elements are jointly optimized by using AMPA and five other existing algorithms.

The rest of the work is arranged as follows. Section 2 explains the CAA model and optimization objective. Section 3 describes the AMPA technique and Section 4 presents the simulation results. Lastly, Section 5 gives the conclusion.

## 2. Antenna Array Model and Problem Formulation

### 2.1. The CAA Model

**Figure 1:**
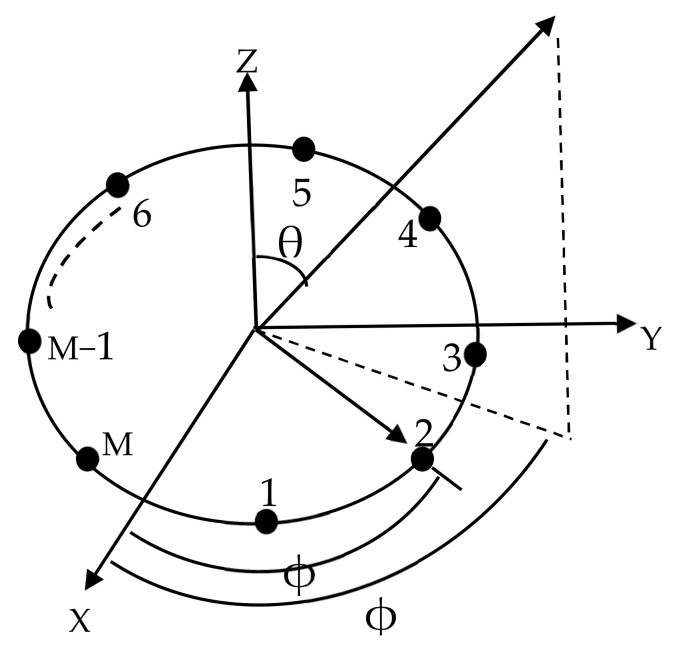
Asymmetric N isotropic circular antenna array (CAA).

As shown in Figure 1, we consider an asymmetric circular antenna array of N isotropic elements with equal excitation and element spacing. The array factor is expressed as [18]:

(1) $$AF(\theta )=\sum_{m=1}^{M}I_{m}\times e^{i(kr.\cos(\theta -\phi_{m})+\alpha_{m})}.$$

(2) $$kr=\frac{2\pi r}{\lambda }\sum_{j=1}^{M}d_{j}.$$

(3) $$\phi_{m}=\left(\frac{2\pi r}{\lambda }\right).\sum_{j=1}^{m}d_{j}.$$

where *AF*, *I_m_*, *d_m_*, and *α_m_* denote the array factor, the excitation, the position of the mth element, and the phase of the antenna array.

### 2.2. Problem Formulation

This work aims at minimizing the SLL of the CAA by jointly optimizing its excitation amplitude and inter-element spacing. Hence, the objective function is given as:

(4) $$\;f=w_{1}\times \underset{\mathrm{SLL}\;\mathrm{supression}}{\underbrace{\left(\max(AF(\theta_{PSL}^{R_{1}}))+\max(AF(\theta_{PSL}^{R_{2}}))\right)}}.$$

where *w*_1_ is the weight, and $\theta_{PSL}^{R_{1}}$ and $\theta_{PSL}^{R_{2}}$ denote the peak SLL suppression angles. The equation suppresses the SLL region of the array pattern; thus, the optimization problem is formulated as:

(5) $$\min\;f(I_{1},I_{2},I_{3},\ldots \ldots ,I_{n},d_{1},d_{2},d_{3},\ldots \ldots ,d_{n}).$$

(6) $$s.t.?\;\;\theta_{PSL}^{R}=\in \max(\left[-\pi ,\theta_{R1}\right]\cup \left[\theta_{R2},\pi \right]).\;$$

(7) $$0\leq I_{m}<1,m=1,2,3,\ldots \ldots ,M\;$$

(8) $$d_{\mathrm{mi}}-d_{m}\geq d_{\min}<d_{\max},m=1,2,3,\ldots \ldots ,M\;$$

where $\left[-\pi ,\theta_{R1}\right]$ and $\left[\theta_{R2},\pi \right]$ are the regions of the SLL suppression. *d*_min_ and *d*_max_ are the minimum and maximum inter-element distances.

## 3. Proposed Algorithm

This section discusses the MPA, and the novel proposed advanced marine predator algorithm’s (AMPA) operation mechanisms.

### 3.1. Marine Predators Algorithm (MPA)

In MPA, the initial solution is randomly generated and uniformly distributed over the search space, keeping in view the upper and lower range of values of the design variables. The fittest solution is nominated as the top predator to construct a matrix called Elite. In this algorithm, both the predator and prey are considered search agents because as the predator is in search of its prey, the prey is also in search of its food.

The optimization phase of the standard MPA is divided into three stages based on the velocity ratio and the entire activity of the predator and prey. The first phase is the exploration stage, and it occurs in the first third of iterations. The mathematical expression is written as:

(9) $$\begin{array}{l} While\;Iteration<\frac{1}{3}\;Max\_Iteration\\ {\vec{Stepsize}}_{j}={\vec{r}}_{b}⊗(\vec{Elite}-{\vec{r}}_{b}⊗{\vec{\mathrm{Pr}ey}}_{j})\;j=1,2,\ldots n\\ {\vec{\mathrm{Pr}ey}}_{j}={\vec{\mathrm{Pr}ey}}_{j}+C\cdot \vec{r}⊗{\vec{Stepsize}}_{j} \end{array}$$

where $r_{b}$ represents the Brownian motion, which is a random number based on normal distribution, and *r* is a uniform random number between 0 and 1. *C* is a constant number that equals 0.5. The second phase of the algorithm commences at the two-third stage of the iteration, while the predator and the prey are moving at the same pace. This can be expressed as:

(10) While 13 Max_Iteration<iteration<23 Max_IterationFor the first half of the populationStepsize→j=r→l⊗(Elitej→−r→l⊗Prey→j) j=1,2,…n2Prey→j=Prey→j+C⋅r→⊗Stepsize→jFor the second half of the populationStepsize→j=r→b⊗(r→b⊗Elitej→−r→l⊗Prey→j) j=n2,…nPrey→j=Elite→j+C⋅AP⊗Stepsize→j

where ${\vec{r}}_{l}$ represents the Lévy movement, which is a random number based on the Lévy distribution. *AP* is the adaptive parameter assigned to control the step size of the predator movement and it is expressed as:

(11) $$AP={\left(1-\frac{Iteration}{Max\_Iteration}\right)}^{\left(2\frac{Iteration}{Max\_Iteration}\right)}$$

The third phase occurs when the iteration is greater than two-thirds of the maximum iteration. This phase is presented as:

(12) $$\begin{array}{l} {\vec{Stepsize}}_{j}={\vec{r}}_{l}⊗({\vec{r}}_{l}⊗\vec{Elite}-{\vec{\mathrm{Pr}ey}}_{j})\;j=1,2,\ldots n\\ {\vec{\mathrm{Pr}ey}}_{j}={\vec{Elite}}_{j}+C\cdot AP⊗{\vec{Stepsize}}_{j} \end{array}$$

Environmental factors, such as the fish aggregating devices (FADs) effects or the eddy formation effect, also cause behavioral changes in the predator. These FADs serve as local optima where the predators can be easily trapped. To escape the FADs and avoid stagnation, the predator or prey needs to make a longer jump, else it will become trapped. The FADs effect is expressed as:

(13) $${\vec{\mathrm{Pr}ey}}_{j}=\left\{\begin{array}{ll} {\vec{\mathrm{Pr}ey}}_{j}+AP\left[{\vec{x}}_{\min}+\vec{r}⊗({\vec{x}}_{\max}-{\vec{x}}_{\min})\right]⊗\vec{u} & if\;r\leq FADs\\ {\vec{\mathrm{Pr}ey}}_{j}+\left[FADs(1-r^{1})+r^{1}\right]({\vec{\mathrm{Pr}ey}}_{r1}-{\vec{\mathrm{Pr}ey}}_{r2}) & if\;r>FADs \end{array}\right.$$

where ${\vec{x}}_{\min}$ and ${\vec{x}}_{\max}$ represent the vectors containing the lower and upper bound of the dimensions. $r^{1}$ is a random number, whereas *r*1 and *r*2 subscripts are random indexes of the prey matrix. $\vec{u}$ is a binary vector that has arrays that include 0 and 1. The probability of the FADs effect equals 0.2.

The steps of MPA can be found in [27].

### 3.2. Advanced Marine Predators Algorithm (AMPA)

The operation mechanisms of the AMPA are as follows:

#### 3.2.1. Initialization

The predator and prey are a matrix formed from the best or fittest randomly generated solution, which are called Elite and Prey, respectively, as conformed to the standard MPA. The format of the Elite and Prey for the AMPA is expressed as:

(14) $$Elite/\mathrm{Pr}ey={\left[\begin{array}{llll} X_{1,1} & X_{1,2} & \ldots \ldots & X_{1,d}\\ X_{2,1} & X_{2,2} & \ldots \ldots & X_{2,d}\\ \vdots & \vdots & \vdots & \vdots \\ X_{n,1} & X_{n,2} & \ldots \ldots & X_{n,d} \end{array}\right]}_{n\times d}$$

where *n* is the population size, *d* is the dimension, and X represents the randomly generated solution.

#### 3.2.2. Optimization Process

The AMPA optimization process is divided into two phases, unlike the standard MPA which has three phases. This algorithm is also subjected to the same environmental factor effects as the standard MPA.

(1) Phase 1: In the first phase, an improved velocity update and position update are introduced to the algorithm. This velocity update takes into consideration both the personal best fitness of individual prey or Elite, and also their global best fitness. This gives room for the expansion of the exploration capacity of the algorithm, unlike the standard MPA that uses only the best fitness. The velocity update used in this technique is expressed mathematically as:

(15) $$\vec{Stepsize_{i,j}}=AP\times \vec{Stepsize_{i,j}}+b_{1}e^{-\beta r_{p}^{2}}(pbest_{i,j}-\mathrm{Pr}ey_{i,j})+b_{2}e^{-\beta r_{g}^{2}}(gbest_{i,j}-\mathrm{Pr}ey_{i,j})$$

where *AP*, *stepsize,* and *β* are the adaptive weight, velocity, and distance sight coefficient, respectively. *b*_1_ and *b*_2_ are learning coefficients. *gbest* and *pbest* symbolize the global and personal best positions, respectively. *r_p_* represents the Euclidean distance between the personal best positions and the current positions of the prey, whereas *r_g_* represents the Euclidean distance between the global best position and the current positions of the prey. The updated Prey is thus expressed as:

(16) $${\vec{\mathrm{Pr}ey}}_{i,j}={\vec{\mathrm{Pr}ey}}_{i,j}+C\times RC\times {\vec{Stepsize}}_{i,j}$$

where *C* is a constant number that equals 0.4, and *RC* is the chaos called the Chebyshev map.

Chebyshev map: This is a one-dimensional chaotic map with an easy and simple to implement iterative equation. It exhibits pseudo-randomness of output sequences, as well as high ergodic property and sensitivity to parameter and initial value [31]. It is mathematically expressed as [31]:

(17) $$x_{s+1}=\cos(k\cos^{-1}(x_{s}))$$

In this work, the Chebyshev map is used to generate chaotic sequences to improve the stepsize, and hence enhance the exploitation and convergence of the algorithm. The chaos introduces randomness generated by a simple deterministic dynamic system called the chaotic system into the algorithm to help the algorithm explore the search space more dynamically and globally without being trapped into local optima. The starting value and the number of iterations used for the Chebyshev map are 0.7 and 100, respectively.

(2) Phase 2: The second phase of this algorithm is made to conform to the last phase of the standard MPA algorithm. At this phase, the predator is assumed to move at a faster pace than the prey, with high exploitation capability, and a low-velocity ratio of 0.1 (v = 0.1). Here, the predator’s movement is Lévy, and the phase is presented as shown in Equation (12).

The mathematical expression for the Lévy movement is given as:

(18) $${\vec{r}}_{l}=0.05*(\frac{N_{randn}\times \delta }{{\left[abs(N_{randn})\right]}^{\frac{1}{\beta }}})$$

(19) $$\delta ={\left\{\frac{\Gamma (1+\beta )\times \sin(\frac{\pi }{2}\times \beta )}{\left[\Gamma (\frac{1+\beta }{2})\times \beta \times 2^{\left(\frac{\beta -1}{2}\right)}\right]}\right\}}^{\frac{1}{\beta }}$$

where $N_{randn}$, *β*, Γ, and *δ* represent the random number, spatial exponent, gamma function, and random variable, respectively. This optimization process is subjected to the same FAD effect as that of the standard MPA expressed in Equation (13).

The basic steps of the AMPA are summarized in the pseudo-code shown in Algorithm 1.
**Algorithm 1.** Pseudo-code of advanced marine predator algorithm (AMPA) (AMPA code)Initialize parameters: m, FADs, C Initialize search agents (Prey) populations *x_i_* = (*x_i_*, *x_i_*, …, *x_i_*) and step size **While** maximum iteration is not met Evaluate the fitness Construct the Elite and Prey matrix using Equation (14) Accomplish memory saving **If** rand < 0.6 Update the stepsize and prey using Equations (15) and (16) **Else** Update prey using Equation (12) **End** (if) Evaluate fitness of the updated prey Save and update the Elite Apply the FADs effect and update using Equation (13) End **while**

## 4. Simulation Results

The performance of the proposed algorithm is evaluated by applying it to the circular antenna array pattern synthesis. The simulation results obtained by the AMPA are compared with five other state-of-art algorithms, which are MPA, arithmetic optimization algorithm (AOA), moth flame optimization (MFO) algorithm, grey wolf optimization (GWO), and IWO. The parameter settings of all algorithms are given in Table 1. The simulations are performed on an AMD A10-5750M APU with a Radeon (tm) HD Graphics personal computer with Windows 10 operating system, 2.5 GHz processor, and 8 GB ram, using MATLAB 2020a. The tuning of the parameter values of the proposed AMPA for CAA pattern synthesis is also performed.

### 4.1. Parameter Value Tuning

**Figure 2:**
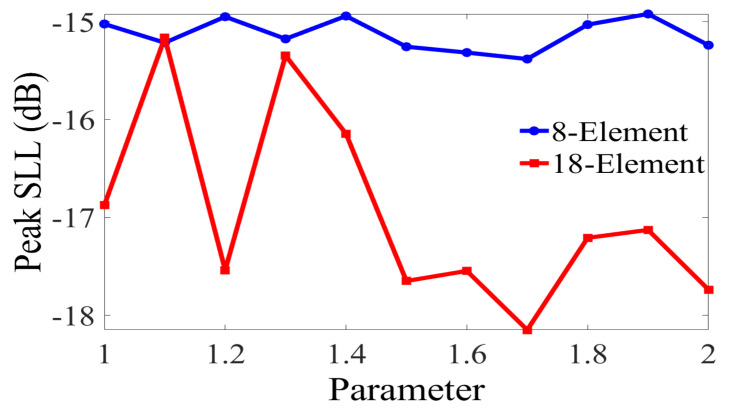
Selection of b1 and b2 for both 8–element and 16–element CAA.

The parameters of AMPA are tuned for antenna optimization. The learning coefficient values ‘*b*_1_ and *b*_2_*′* are very sensitive parameters that play a great role in improving the exploration capability of the algorithm; therefore, it is being tuned. We vary the values of *b*_1_ and *b*_2_ from 1.0 to 2.0, at a step size of 0.1, while using the AMPA to solve the formulated optimization problem. The test was repeated 50 times to eliminate random bias, and the best results are presented in Figure 2. We observed that the 8-CAA and 18-CAA elements obtained an optimal peak SLL at a value of 1.7. Therefore, we choose to set the values of the learning coefficients to 1.7 for the CAA synthesis in this research work.

### 4.2. Simulation Result for the CAA Pattern Synthesis

In this subsection, the proposed AMPA and other algorithms stated in Table 1 are applied to solve the formulated CAA optimization problem in Section 2. The maximum number of iterations, population size, and number of runs are set to 100, 20, and 50, respectively, for all the algorithms. The simulation is performed on four different examples of CAA, which are 8-element, 10-element, 12-element, and 18-element CAA. To cater for the mutual coupling between the elements and avoid possible grating lobes, the inter-element spacing was constrained to range between 0.252 λ and 0.999 λ. The excitation amplitude ranged from 0 to 1. The best optimization results for each algorithm are recorded in this work.

#### 4.2.1. Beam Pattern Synthesis of 8-Element CAA

**Figure 3:**
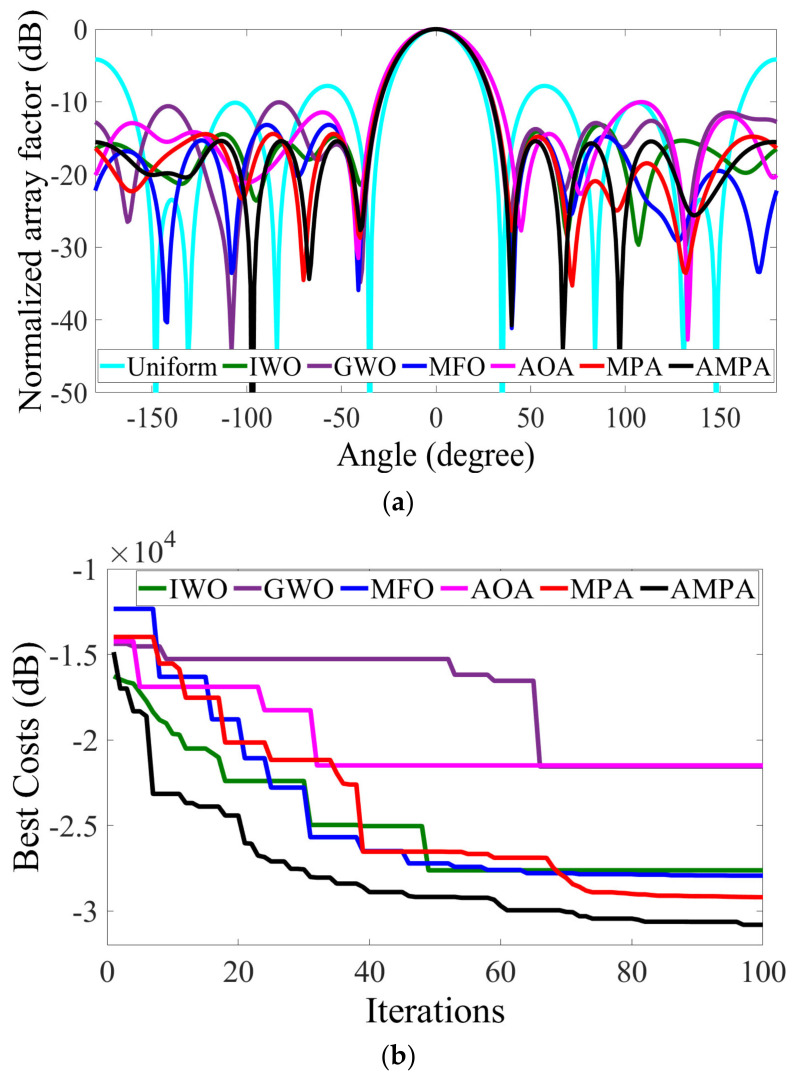
8−element CAA. (**a**) Radiation patterns obtained by different algorithms for reducing the PSLL. (**b**) Convergence rates of different algorithms for reducing the PSLL.

Table 2 shows the peak SLL, FNBW, circumference, and total computational time obtained by different optimization algorithms for the synthesis of 8-element CAA. AMPA obtained the lowest SLL of −15.3811 dB, which is 0.9659 dB, 5.3435 dB, 2.2027 dB, 5.3183 dB, 2.2158 dB, and 11.2109 dB lower than MPA, AOA, MFO, GWO, and IWO, respectively. The optimized excitation amplitude and element spacing obtained by AMPA is displayed in Table 3. Though AMPA achieved the best SLL, it was also able to acquire an FNBW of 81.00°, which is narrower than that of AOA and GWO. The radiation pattern and convergence rate of this analysis are shown in Figure 3a,b. The convergence curve clearly shows how AMPA is able to overcome being stuck in local optimum, and thus achieve the best SLL suppression results.

#### 4.2.2. Beam Pattern Synthesis of 10-Element CAA

**Figure 4:**
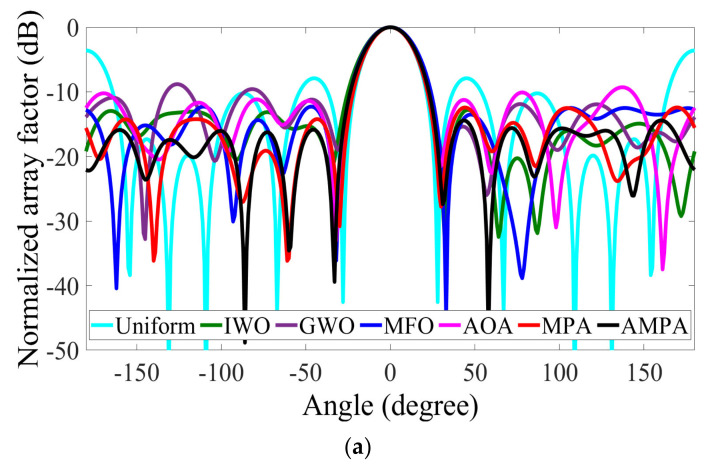

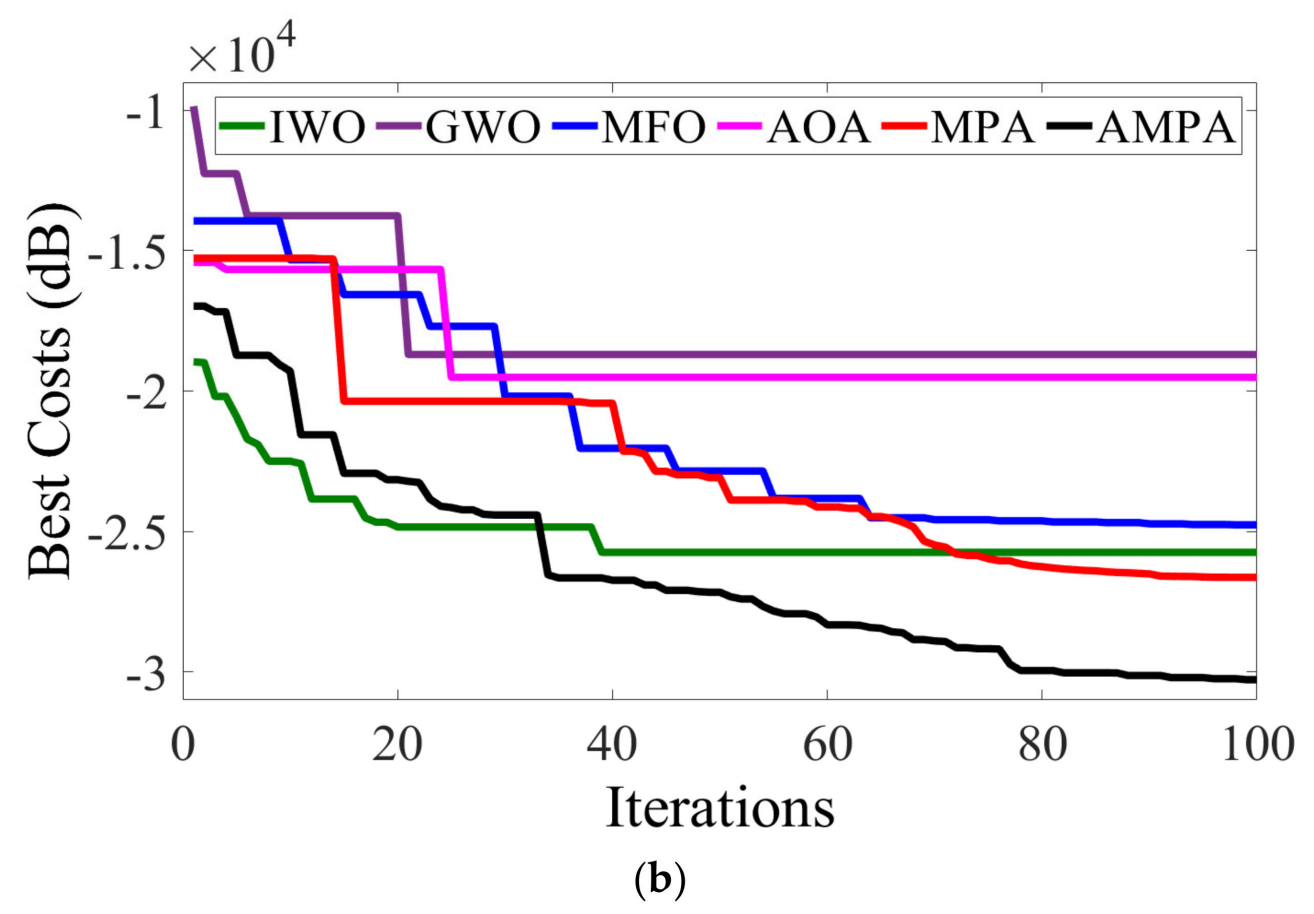
10−element CAA. (**a**) Radiation patterns obtained by different algorithms for reducing the PSLL. (**b**) Convergence rates of different algorithms for reducing the PSLL.

**Figure 5:**
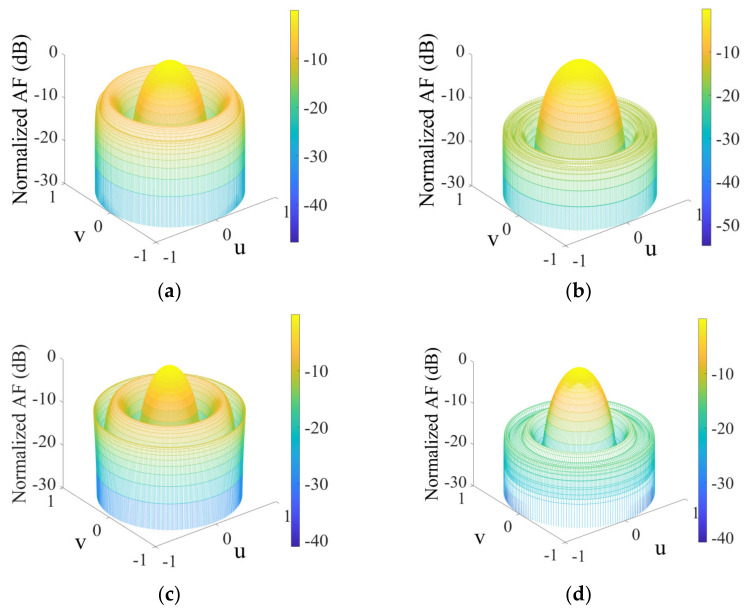
3D radiation patterns of CAA. (**a**) 8−element CAA uniform array; (**b**) 8−element CAA for AMPA; (**c**) 10−element CAA uniform array; (**d**) 10−element CAA for AMPA.

The peak SLL values of −14.4185 dB, −12.4175 dB, −9.2885 dB, −12.2772 dB, −8.8086 dB, and −12.7904 dB are achieved by IWO, GWO, MFO, AOA, MPA, and AMPA, respectively, as shown in Table 4. The proposed AMPA obtained the best SLL suppression with an FNBW of 64° and an aperture length of approximately 6.00 λ. MPA obtained the narrowest FNBW, followed by the AOA and other algorithms, whereas IWO had the largest FNBW. With the aid of the velocity update mechanism, it became easy for the AMPA to escape local optimum, while the chaos improves its ability to exploit a larger search space. This is obvious from the radiation pattern and convergence plot depicted in Figure 4a,b. Table 5 shows the optimized parameters used to plot the radiation pattern of AMPA for the 10-element CAA. The 3D beam pattern reflecting the level of improvement between the uniform and AMPA-improved CAA for 8-, and 10-element CAA is shown in Figure 5a–d.

#### 4.2.3. Beam Pattern Synthesis of 12-Element CAA

**Figure 6:**
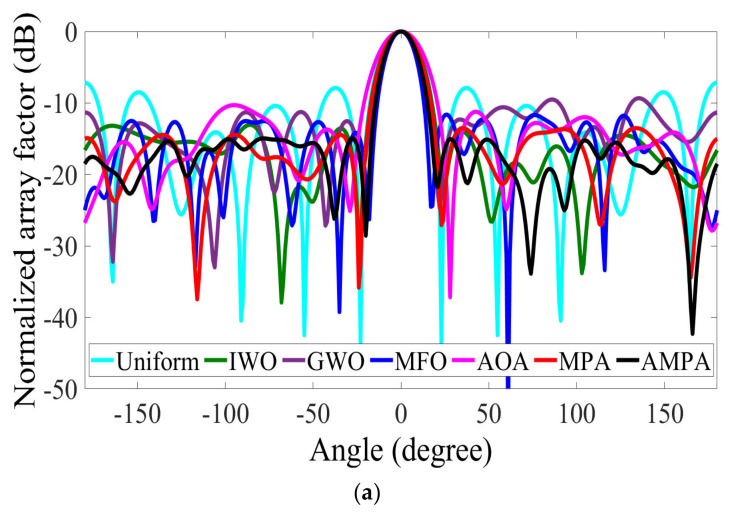

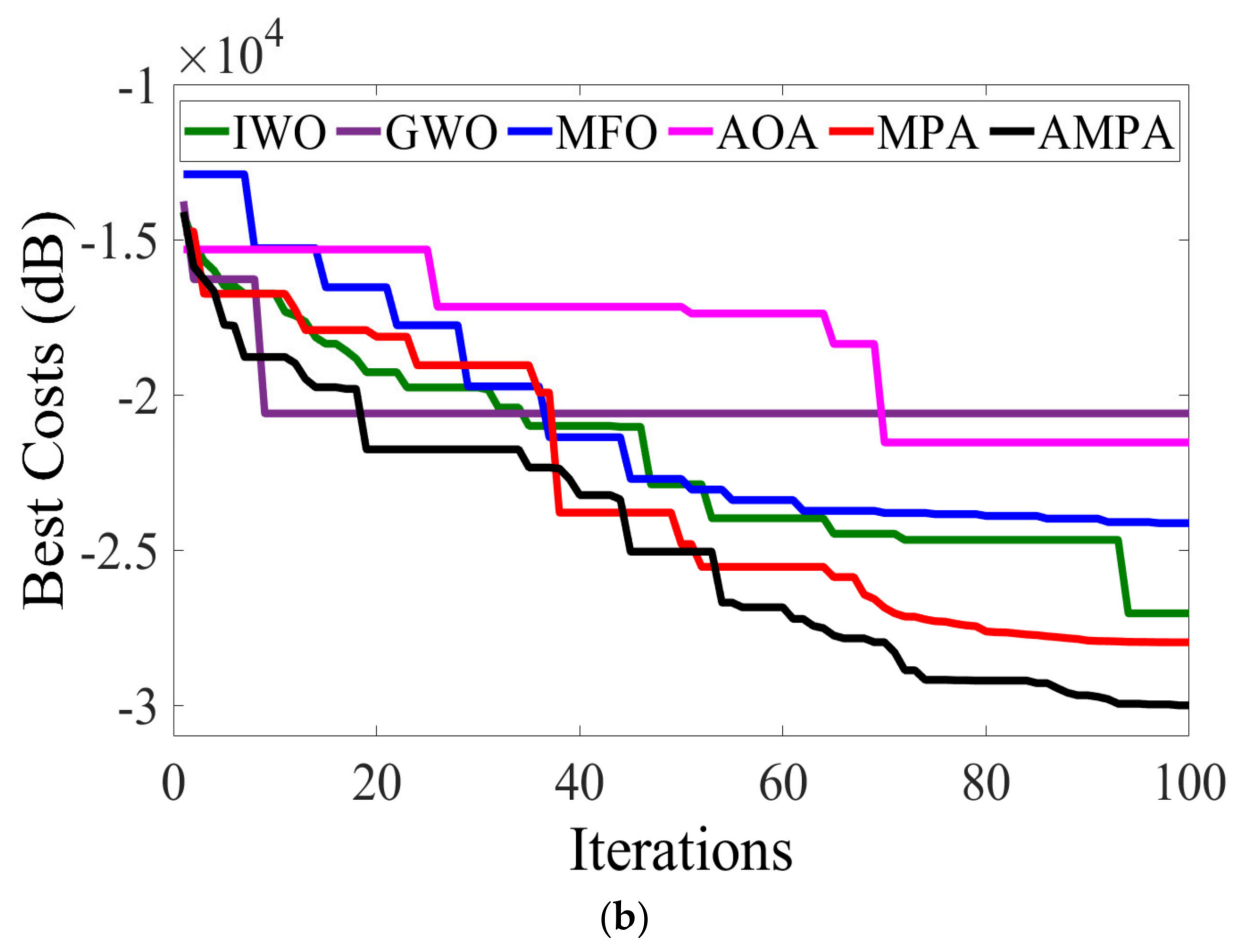
12−element CAA. (**a**) Radiation patterns obtained by different algorithms for reducing the PSLL. (**b**) Convergence rates of different algorithms for reducing the PSLL.

The numerical results in terms of the PSLL, FNBW, circumference, and total computational time are recorded in Table 6. The solutions I and d, obtained by AMPA for this optimization goal, are recorded in Table 7. It can be seen from Table 6 and Figure 6a that the proposed AMPA not only attained the lowest peak SLL as compared with the other benchmark algorithms, but also had an FNBW of 41.00°, which is narrower than that of the uniform array by 5°. This proves that the AMPA has an excellent performance. MFO achieved the highest aperture length of 9.35 λ and the narrowest FNBW. GWO had the highest peak SLL, followed by AOA and MFO. The computational time of both AMPA and MPA compares favorably. The convergence curve of the algorithms is shown in Figure 6b.

#### 4.2.4. Beam Pattern Synthesis of 18-Element CAA

**Figure 7:**
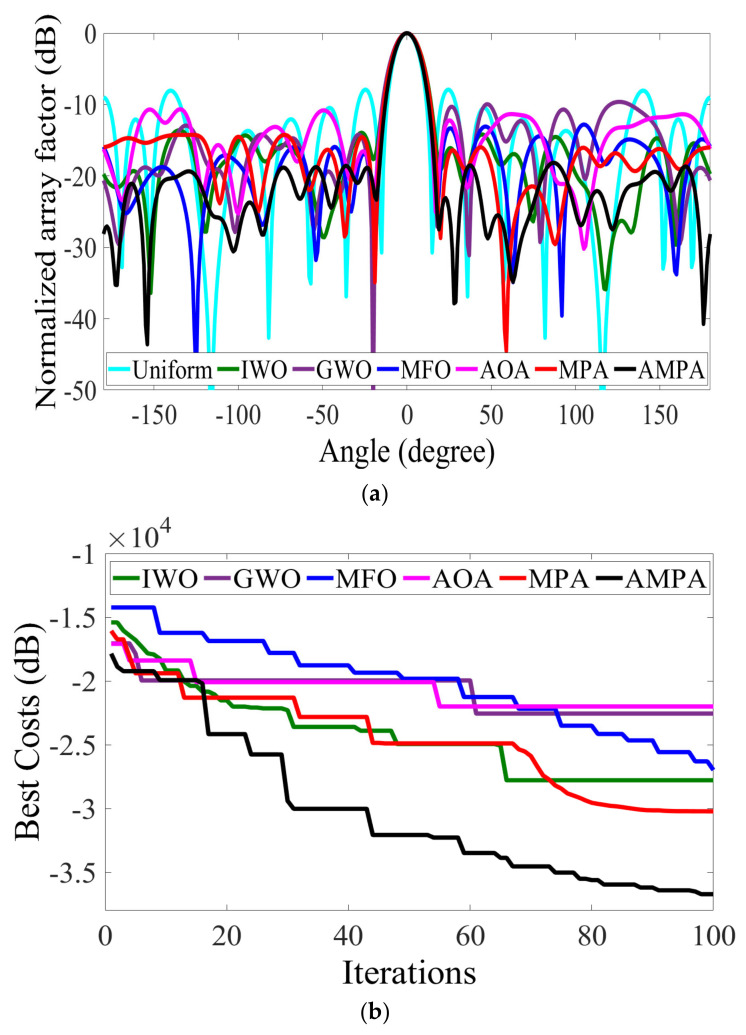
18−element CAA. (**a**) Radiation patterns obtained by different algorithms for reducing the PSLL. (**b**) Convergence rates of different algorithms for reducing the PSLL.

**Figure 8:**
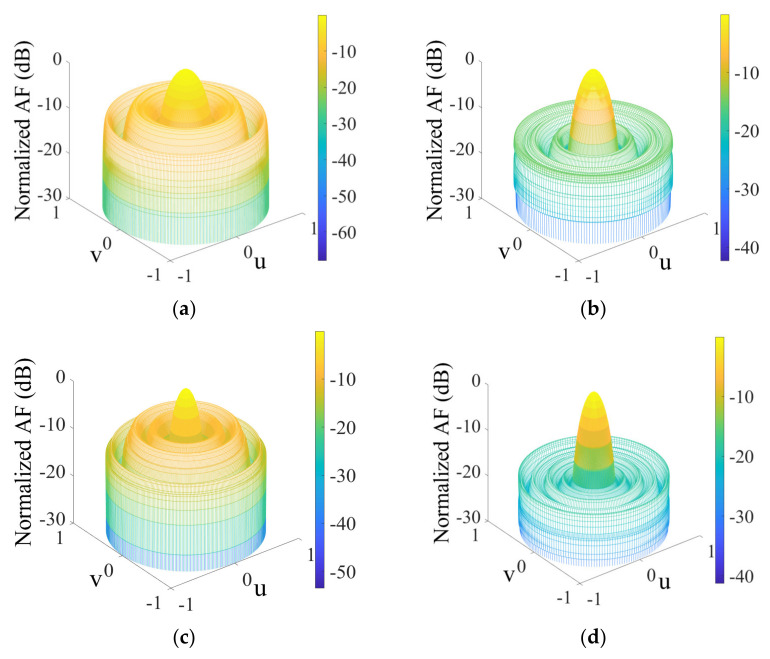
3D radiation patterns of CAA. (**a**) 12−element CAA uniform array; (**b**) 12−element CAA for AMPA; (**c**) 18−element CAA uniform array; (**d**) 18−element CAA for AMPA.

**Figure 9:**
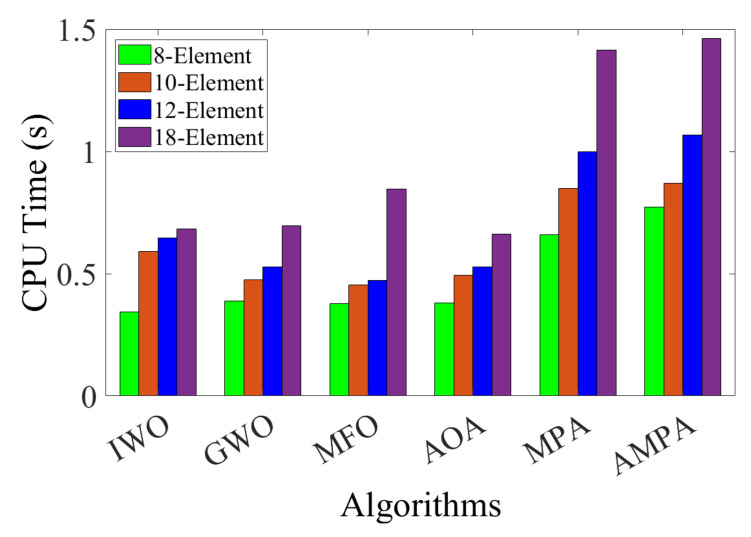
Bar graph for the computational time obtained by the algorithms for each CAA example.

The optimization result comparison of the 18-element CAA is made in Table 8, and the array pattern is shown in Figure 7a. According to the result, it is observed that AMPA attained a peak SLL of −18.1481 dB, which obviously supersedes MPA, AOA, MFO, GWO, IWO, and uniform array by −3.9114 dB, −7.4804 dB, −5.3536 dB, −8.5261 dB, 4.5531 dB, and −10.2312 dB, respectively. The solutions obtained by AMPA are recorded in Table 9. The capability of the AMPA is reflected in the radiation pattern and convergence curve (Figure 7). Though AMPA covered the largest circumference, it still attains excellent SLL reduction with an FNBW of 37.00°. With the low SLL obtained by AMPA, it is easy to avoid the interference with the other system operating in the same frequency band. Figure 8a–d depict the 3D beam pattern that compares the 12- and 18-element CAA’s uniform and AMPA-improved beam patterns. The computational time obtained by each algorithm for optimizing the 8-, 10-, 12-, and 18-element CAA is presented in Figure 9. It can be seen that the AMPA has the highest computational time throughout the entire simulation. This is owed to the algorithm’s computational complexity, as it requires more mathematical steps to achieve the given optimal results than others. Figure 9 also shows that each algorithm’s computational time increases as the CAA’s elements increases.

## 5. Conclusions

This study proposes a new method called advanced marine predator algorithm (AMPA) for the synthesis of CAA. The proposed method is easy to implement, and it effectively optimizes both excitation current and element-spacing simultaneously. It is computationally fast and has a better convergence rate than MPA, AOA, MFO, GWO, and IWO, which it was compared with; thus, it proves to be more efficient in the CAA optimization. The optimal peak SLL values of −15.3811 dB, −14.4185 dB, −14.9518 dB, and −18.1481 dB, obtained by the AMPA for 8-, 10-, 12-, and 18- element CAA, respectively, verified the efficacy of the algorithm to easily escape local optima and obtain better optimization solutions. With the information provided in this research, a practical antenna array design can be made to meet the needs of the optimal communication system. Further improvement can be made to this algorithm by exploring other chaotic sequences to enhance its optimization potential.

## Figures and Tables

**Table 1:** Parameter settings of the algorithms.

Algorithm	Parameters	Values
AMPA	b_1_ and b_2_	1.7
	FADs and C	[0.2, 0.4]
MPA	FADs and C	[0.2, 0.5]
AOA	α and μ	[5, 0.499]
MFO	Spiral constant	1
	Convergence constant	[−1, −2]
	Random factor	[−1, 1]
IWO	Exponent	2
	Minimum and maximum number of seeds	[0, 5]
	Initial and final SD	[0.01, 0.1]
GWO	Control parameter	[2, 0]

**Table 2:** Results of 8-element CAA obtained by different algorithms for PSLL minimization.

Algorithm	Peak SLL (dB)	FNBW (°)	Circumference (λ)	CPU Time (s)
Uniform	−4.1702	70.00	3.75	0.00
IWO	−13.1653	80.00	4.49	0.34
GWO	−10.0628	80.00	4.45	0.39
MFO	−13.1784	81.00	4.41	0.38
AOA	−10.0376	86.00	4.40	0.38
MPA	−14.4152	80.00	4.52	0.66
AMPA	−15.3811	80.00	4.55	0.77

**Table 3:** Amplitude (I) and element spacing (d) obtained by using AMPA for 8-element CAA PSLL minimization.

Element	1	2	3	4	5	6	7	8
I	0.8111	0.4236	0.9577	0.9793	0.0435	0.3654	0.8533	0.0962
d (λ)	0.3137	0.8028	0.8627	0.6000	0.3684	0.4822	0.7883	0.3272

**Table 4:** Optimization results of 10-element CAA obtained by different algorithms for peak sidelobe level (PSLL) minimization.

Algorithm	Peak SLL (dB)	FNBW (°)	Circumference (λ)	CPU Time (s)
Uniform	−3.5975	56.00	4.75	0.00
IWO	−12.7904	67.00	5.87	0.59
GWO	−8.8086	63.00	5.97	0.47
MFO	−12.2772	65.00	5.83	0.45
AOA	−9.2885	62.00	5.75	0.49
MPA	−12.4175	60.00	6.00	0.85
AMPA	−14.4185	64.00	6.00	0.87

**Table 5:** Amplitude (I) and element spacing (d) obtained by using AMPA for 10-element CAA PSLL minimization.

Element	1	2	3	4	5	6	7	8	9	10
I	0.9540	0.4040	0.3468	0.9940	0.9864	0.3329	0.5148	0.1317	0.9996	0.3978
d (λ)	0.2920	0.9990	0.4042	0.9990	0.5748	0.9509	0.5501	0.4142	0.4803	0.3313

**Table 6:** Optimization results of 12-element CAA obtained by different algorithms for PSLL minimization.

Algorithm	Peak SLL (dB)	FNBW (°)	Circumference (λ)	CPU Time (s)
Uniform	−7.165	46.00	5.75	0.00
IWO	−13.0541	48.00	7.23	0.64
GWO	−9.34022	44.00	7.66	0.53
MFO	−11.6163	35.00	9.35	0.47
AOA	−10.3039	57.00	5.97	0.53
MPA	−13.5153	47.00	7.34	1.00
AMPA	−14.9518	41.00	9.15	1.07

**Table 7:** Amplitude (I) and element spacing (d) obtained by using AMPA for 12-element CAA PSLL minimization.

Element	1	2	3	4	5	6
I	0.9993	0.7689	0.0865	0.6451	0.9850	0.9998
d (λ)	0.6822	0.9854	0.9781	0.9981	0.6357	0.4636
Element	7	8	9	10	11	12
I	0.8172	0.8431	0.0100	0.8646	0.5589	0.9988
d (λ)	0.4424	0.9990	0.3813	0.9463	0.9515	0.6832

**Table 8:** Optimization results of 18-element CAA obtained by different algorithms for PSLL minimization.

Algorithm	Peak SLL (dB)	FNBW (°)	Circumference (λ)	CPU Time (s)
Uniform	−7.9169	30.00	8.75	0.00
IWO	−13.5950	40.00	9.15	0.68
GWO	−9.6220	38.00	9.17	0.70
MFO	−12.7945	37.00	9.07	0.85
AOA	−10.6677	36.00	9.02	0.66
MPA	−14.2367	39.00	9.16	1.41
AMPA	−18.1481	37.00	10.69	1.46

**Table 9:** Amplitude (I) and element spacing (d) obtained by using AMPA for 18-element CAA PSLL minimization.

Element	1	2	3	4	5	6	7	8	9
I	0.9215	0.6189	0.5579	0.3879	0.0850	0.8766	0.8956	0.6880	0.9204
d (λ)	0.3028	0.4996	0.9128	0.6433	0.7766	0.5094	0.9317	0.4235	0.2744
Element	10	11	12	13	14	15	16	17	18
I	0.8215	0.7992	0.6829	0.7112	0.3970	0.4189	0.3077	0.8632	0.7655
d (λ)	0.3560	0.4245	0.9459	0.6335	0.7798	0.5515	0.9937	0.4096	0.3166
